# An uncommon presentation of hyperhomocysteinemia and vitamin B_12_ deficiency: a case report

**DOI:** 10.1186/s13256-019-1988-9

**Published:** 2019-02-18

**Authors:** Vinay Kapur, Sanjay D’Cruz, Ravinder Kaur

**Affiliations:** 1 Department of General Medicine, Dr HS Judge Institute of Dental Sciences & Hospital, Panjab University, Chandigarh, India; 2 Department of General Medicine, Government Medical College & Hospital, Chandigarh, India; 3 Department of Radiodiagnosis & Imaging, Government Medical College & Hospital, Chandigarh, India

**Keywords:** Hyperhomocysteinemia, Thrombophilia, Venous thrombosis, Vitamin B_12_

## Abstract

**Introduction:**

Cerebral venous thrombosis is relatively rare and characterized by a wide spectrum of clinical features. It is more common in young adults with women affected more than men. The diagnosis of cerebral venous thrombosis is easier nowadays due to easy access to advanced neuroimaging techniques. Abnormalities in thrombophilic profile are associated with enhanced risk of cerebral venous thrombosis. It has varied etiologies such as hypercoagulable states, infection, dehydration, pregnancy, and substance abuse. Hyperhomocysteinemia is found to be closely associated with an enhanced risk of cerebral venous thrombosis.

**Case presentation:**

Here we report a case of cerebral venous thrombosis secondary to hyperhomocysteinemia caused by vitamin B_12_ deficiency in a 32-year-old Indo-Aryan man. A detailed coagulation workup led us to find the etiology of cerebral venous thrombosis in this patient who followed a strict vegetarian diet and had vitamin B_12_ deficiency leading to hyperhomocysteinemia.

**Conclusion:**

There are conflicting reports in the literature about the association of hyperhomocysteinemia, B_12_ deficiency, and cerebral venous thrombosis but some reports point to a significant association. We conclude that further studies with a large sample size are required to analyze the effect of hyperhomocysteinemia and low vitamin B_12_ on the risk of cerebral venous thrombosis.

## Introduction

Cerebral venous thrombosis represents 0.5–3% of all cases of stroke affecting mainly the younger population with an incidence of 3–4 per million in adults [1]. It is a severe thrombotic manifestation resulting in marked disability and has a tendency to recur. It has varied etiologies such as hypercoagulable states, infection, dehydration, pregnancy, drugs such as oral contraceptive pills and substance abuse. This case report highlights the importance of hyperhomocysteinemia (Hhcy) secondary to vitamin B_12_ deficiency leading to cerebral venous thrombosis. The patient mentioned here developed B_12_ deficiency as he followed a strict vegetarian diet and had poor socio-economic status. This case report is significant in the present era when people around the world are changing dietary habits and shifting to a vegan diet which is likely to increase the prevalence of vitamin B_12_ deficiency and subsequently Hhcy. Therefore, it is prudent to include vitamin B_12_ and homocysteine levels in workups of patients presenting with cerebral venous thrombosis.

## Case presentation

**Fig. 1:**
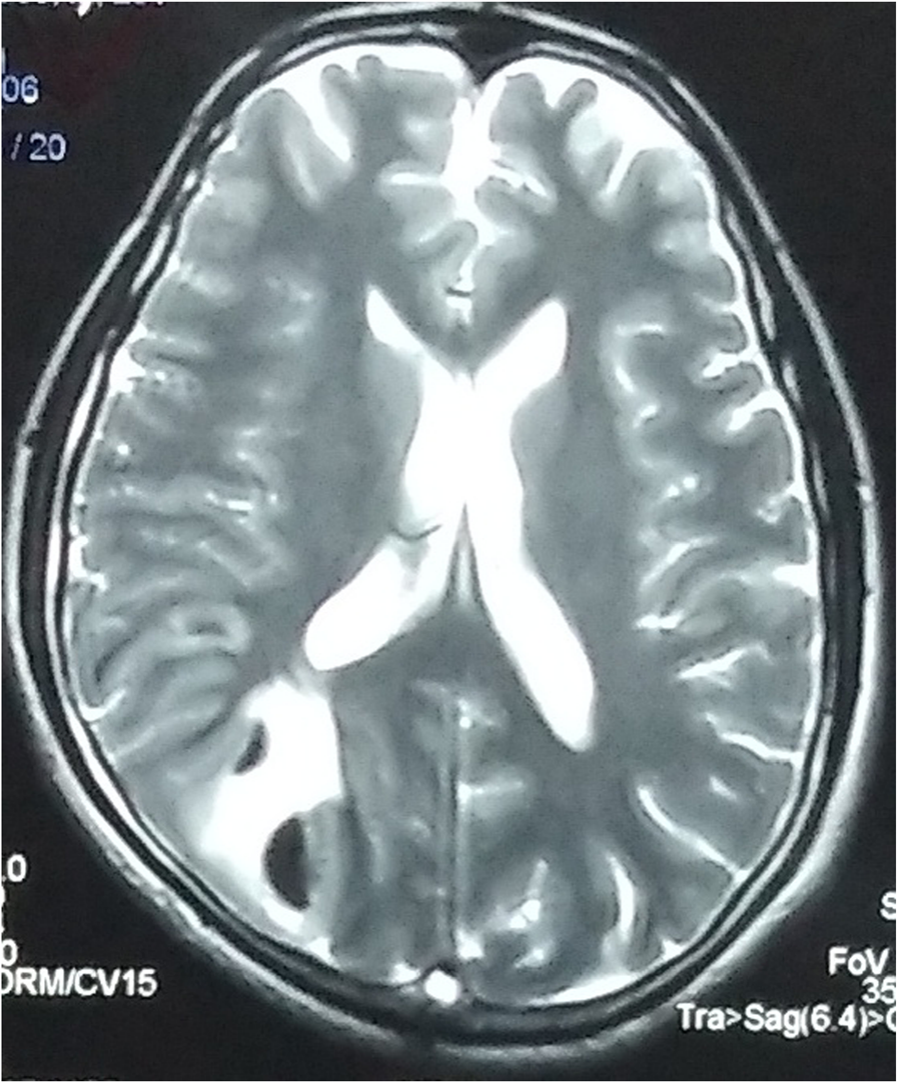
T2-weighted axial images of the brain show mixed signal intensity lesion in right posterior parieto-occipital lobe with areas of hypointensity

**Fig. 2:**
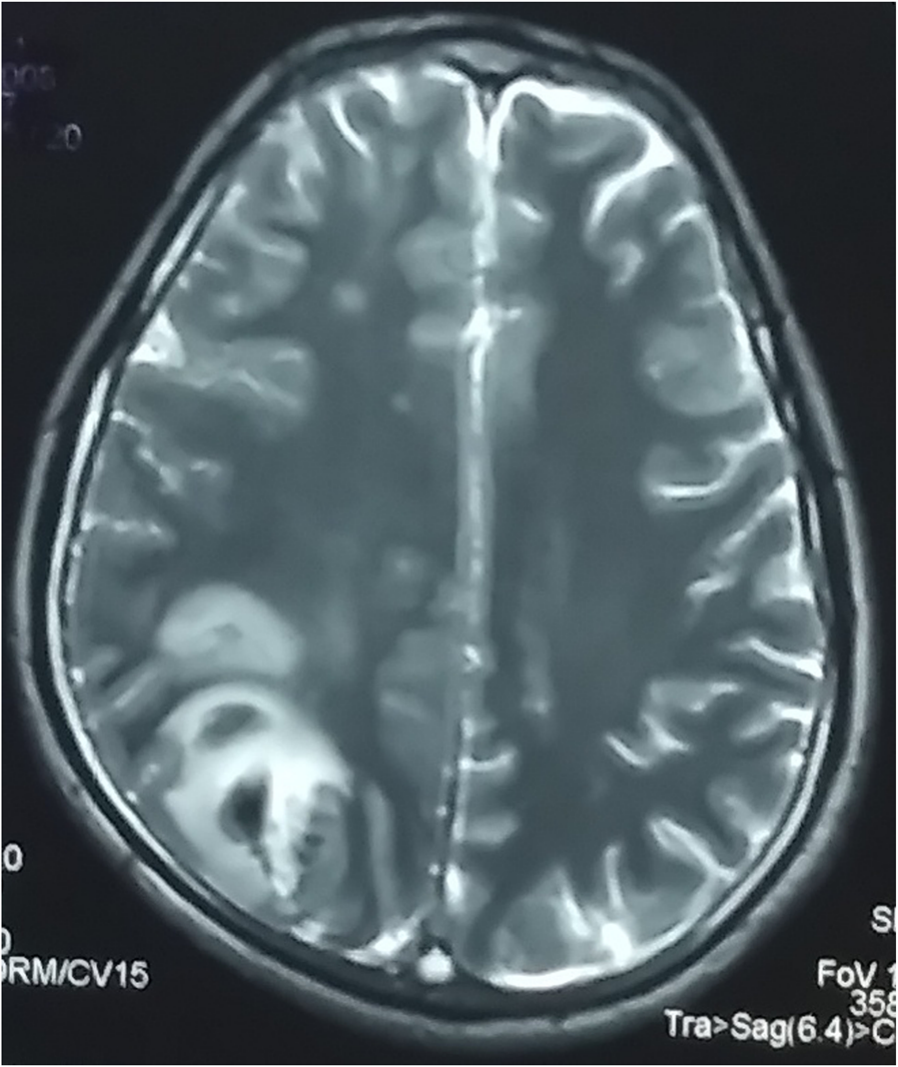
T2-weighted axial images of the brain show mixed signal intensity lesion in right posterior parieto-occipital lobe with areas of hypointensity

**Fig. 3:**
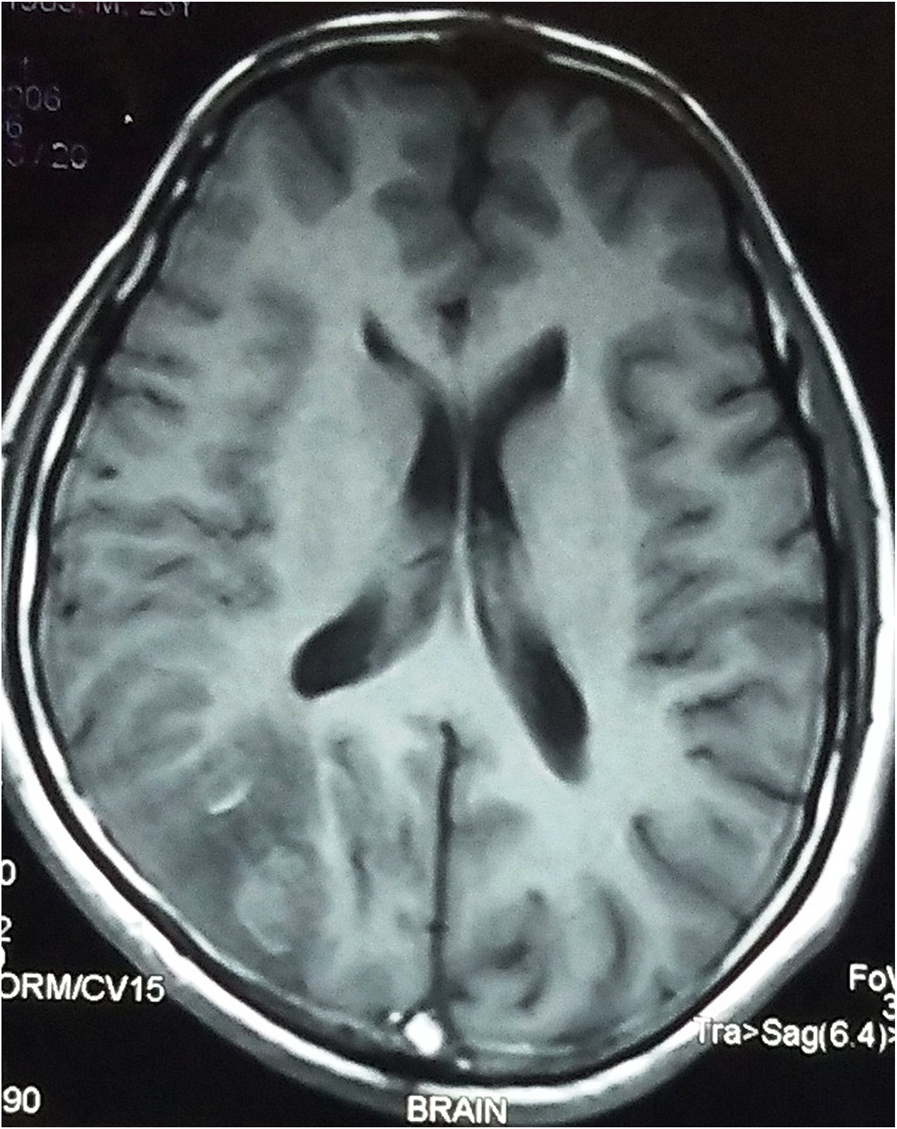
T1-weighted axial images at the same levels show the areas of hyperintensity in the above lesion due to hemorrhage. The normal flow void in the posterior part of superior sagittal sinus is replaced by a hyperintense signal on both T1-weighted and T2-weighted images due to thrombosis

**Fig. 4:**
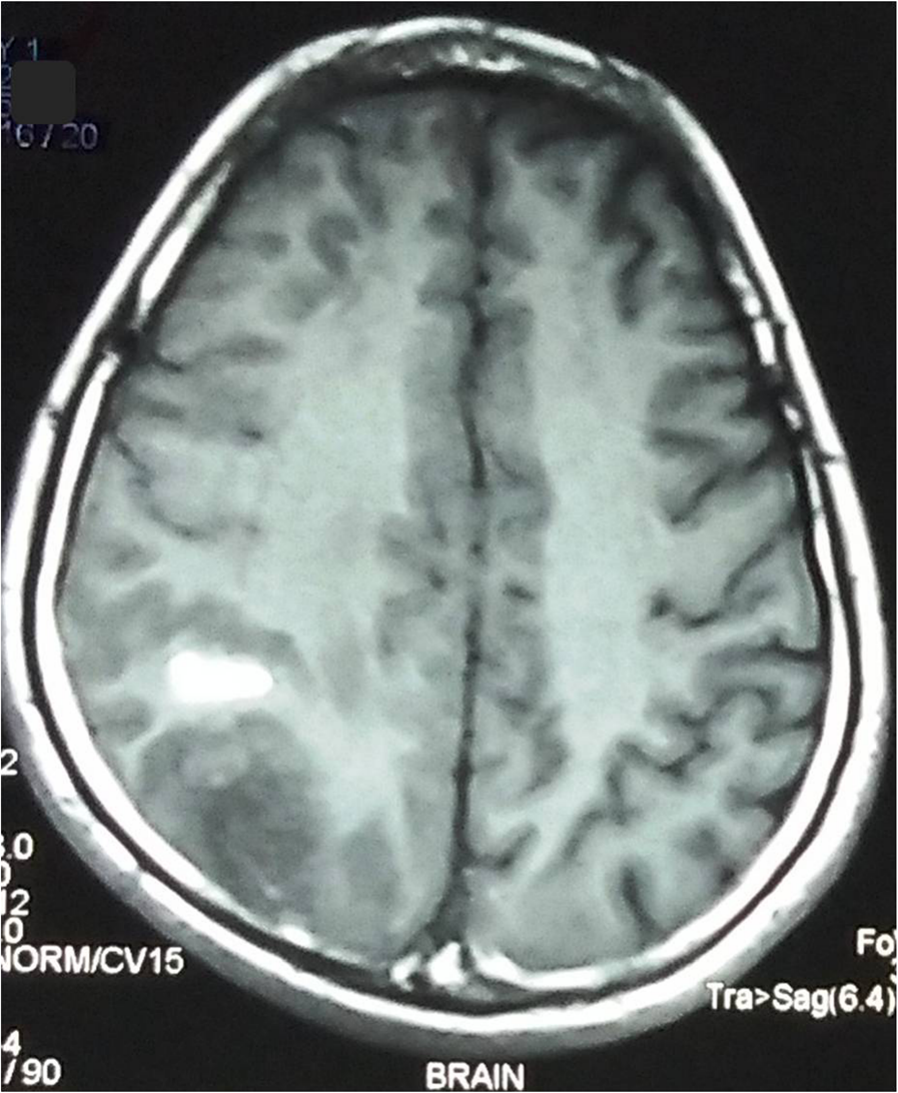
T1-weighted axial images at the same levels show the areas of hyperintensity in the above lesion due to hemorrhage. The normal flow void in the posterior part of superior sagittal sinus is replaced by a hyperintense signal on both T1-weighted and T2-weighted images due to thrombosis

**Fig. 5:**
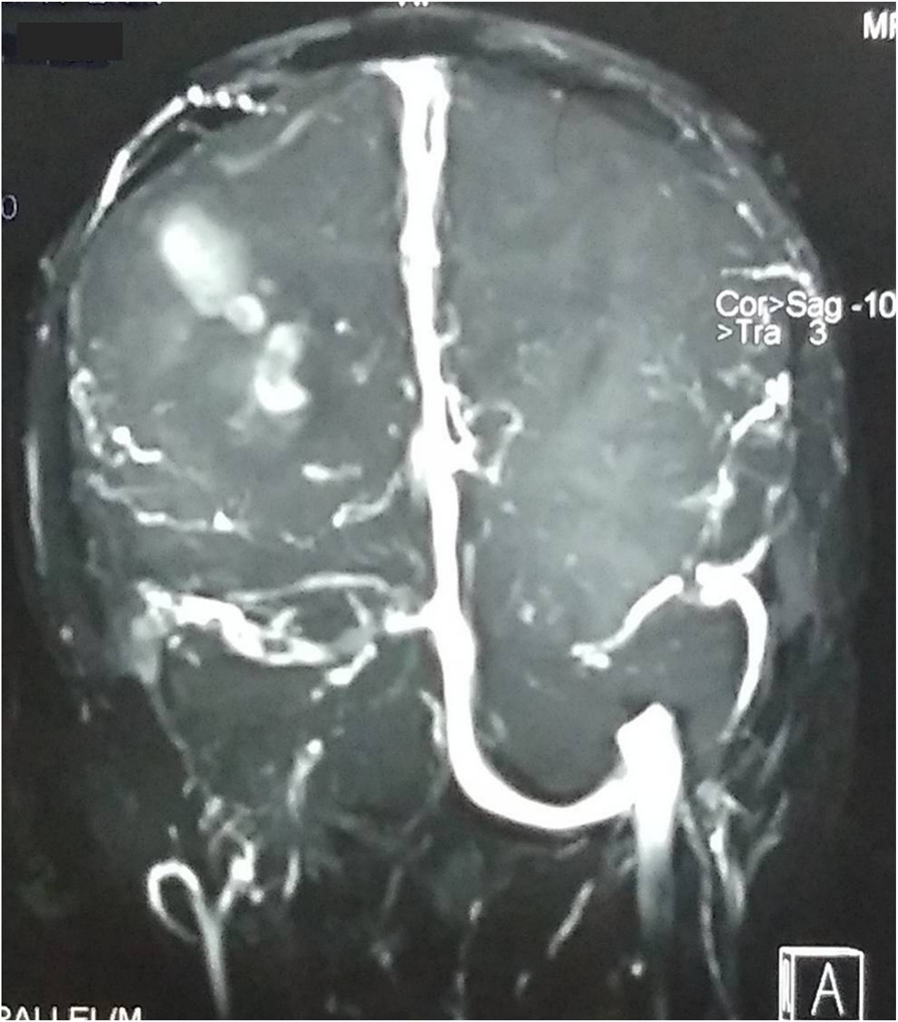
Coronal magnetic resonance venography image shows loss of flow-related signal in transverse and sigmoid sinus due to thrombosis

A 32-year-old Indo-Aryan man presented to our emergency department with history of frequent vomiting, moderate to severe headache and giddiness for past 5 days. He also developed weakness of the right side of his body along with altered sensorium over last 24 hours prior to presentation. There was also history of one episode of generalized tonic-clonic seizures prior to onset of weakness. His past medical history was not suggestive of any major illness/drug treatment. His family history was non-contributory and he had no addictions. He was afebrile with pulse 86/minute and blood pressure of 126/74 mmHg. On neurological examination, he was drowsy and was responding poorly to verbal commands. He was having hypertonia and grade III power in his right upper limb and lower limb. Deep tendon reflexes were mildly exaggerated and Babinski sign was positive on right side. His chest, abdomen, and cardiovascular system examination were unremarkable. His preliminary blood examination revealed macrocytic anemia with hemoglobin (Hb) of 11.4 g/dl and mean corpuscular volume (MCV) of 110 fl. Peripheral blood film showed macrocytes and macro-ovalocytes with hypersegmented neutrophils. He had low serum cobalamin levels 68 pg/ml (200–600) with normal folate levels. Test for anti-intrinsic factor antibodies was negative and there was no evidence of gastric atrophy on stomach biopsy. Cerebrospinal fluid (CSF) examination was normal along with negative immunological profile: antinuclear antibodies (ANA), antineutrophil cytoplasmic antibodies (ANCA), lupus anticoagulant and antiphospholipid antibodies. A detailed thrombophilic workup showed normal prothrombin time 12.8 seconds (11.4–13.7), activated partial thromboplastin time 32.6 seconds (27.8–41.8), protein C 106% (70–140%), protein S 98% (70–140%), and antithrombin III 88% (80–120%). His fibrinogen levels were normal and assays for factor V Leiden and prothrombin gene mutation were negative. The only abnormality was raised fasting total serum homocysteine levels of 36 μmol/l (5.0–13.9). A non-contrast computed tomography (CT) scan of his head was inconclusive. Magnetic resonance imaging (MRI) of his brain showed mixed signal intensity lesion in right posterior parieto-occipital lobe with areas of hypointensity in T2-weighted images (Figs. 1 and 2). T1-weighted axial images at the same levels showed areas of hyperintensity in the above lesion due to hemorrhage (Figs. 3 and 4). Magnetic resonance (MR) venography showed no signal in right transverse and sigmoid sinus due to venous thrombosis (Fig. 5). A diagnosis of cerebral venous thrombosis due to Hhcy secondary to cobalamin deficiency was made.

He was treated with sodium valproate 20 mg/kg administered intravenously followed by orally administered valproate 20 mg/kg in two divided doses which was gradually escalated on follow-up visits. He was also administered cerebral decongestants, initially mannitol 100 ml 8 hourly for 2 days followed by orally administered glycerol 30 ml 8 hourly for 1 week. In addition, he was administered parenteral cyanocobalamin 1000 μg once daily for 7 days along with low molecular weight heparin. Gradually he regained sensorium, his power improved, and he was discharged on orally administered sodium valproate, warfarin, and methylcobalamin. On subsequent monthly follow-ups, his international normalized ratio (INR) was regularly monitored and maintained between 2.0 and 3.0. He remained seizure free thereafter and was continued on sodium valproate 750 mg twice a day. On neurological examination he showed significant improvement, initially he was walking with support and later he was fully ambulatory on his own. Repeat investigations done at 6 months after stopping anticoagulants showed normal serum cobalamin 364 pg/ml (200–600) and fasting total homocysteine levels 8.4 μmol/l (5.0–13.9). The rest of the thrombophilia profile was within normal limits.

## Discussion

**Fig. 6:**
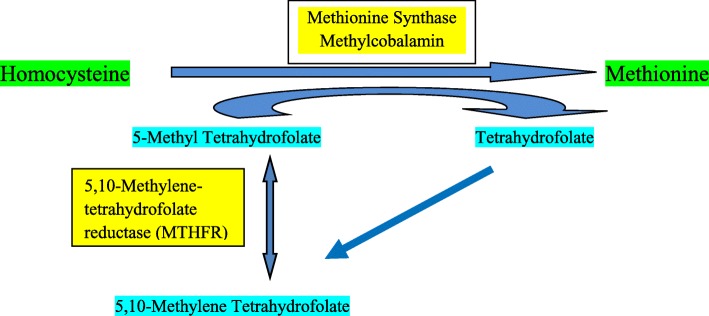
Pathways of homocysteine metabolism

This case report describes an unusual association between Hhcy, low vitamin B_12_ levels and cerebral venous thrombosis as our patient had no other apparent risk factor for venous sinus thrombosis. The prevalence of Hhcy in the general population is approximately 1%, although it may vary from place to place [2]. Hhcy can be caused by genetic and acquired factors, including folate and cobalamin deficiency and methylcobalamin acts as a cofactor with enzyme methionine synthase required for remethylation of homocysteine to methionine. During remethylation, 5-methyltetrahydrofolate, formed from 5,10-methylenetetrahydrofolate in the presence of 5,10-methylenetetrahydrofolate reductase (MTHFR), is converted to tetrahydrofolate (Fig. 6). Hhcy is known to increase arterial thrombotic events in coronary, cerebral, and peripheral arteries [3–5]. Studies have also shown increased risk of venous thrombotic events associated with Hhcy [6, 7]. The main hypotheses put forward to explain the pathogenesis of thrombotic events resulting from increased homocysteine levels include injury to vascular endothelium and adverse effects on clotting cascade. It can lead to increased platelet aggregation, increased low-density lipoprotein (LDL) as well as LDL oxidation, abnormalities of fibrinolysis including decreased activity of antithrombin III and protein C, increased smooth muscle proliferation, activation of inflammatory pathways and thrombocyte dysfunction [8, 9]. Our patient had cerebral venous thrombosis secondary to Hhcy caused by vitamin B_12_ deficiency as he followed a strict vegetarian diet, was from a low socio-economic stratum and apparently had no other risk factors for venous thrombosis. There have been very few studies in the literature which have shown this association. Martinelli *et al*. showed in their study that Hhcy is associated with fourfold increased risk of cerebral venous thrombosis [10]. Patil *et al*. showed that Hhcy was a causative factor for cerebral venous thrombosis in 24% of their patients [11]. Tufano *et al*. reported that frequency of Hhcy was not significantly different between patients with cerebral venous thrombosis and controls [12]. Investigations in our patient revealed low serum B_12_ and Hhcy. On review of the literature, few studies have looked at the issue of B_12_ deficiency and increased thrombotic tendency. Remacha *et al*. indicated that serum vitamin B_12_ levels were moderately related to thromboembolism and vitamin B_12_ deficiency was common among patients with Hhcy and thrombosis [13]. In another study by the same authors, multivariate analysis demonstrated that vitamin B_12_ deficiency was a significant risk factor for arterial thrombosis but when Hhcy was included in the analysis, Hhcy was found to be to be responsible for thrombosis and it was concluded that as a consequence of Hhcy, patients with acquired vitamin B_12_ deficiency had a high risk of thrombosis [14]. Soltani *et al*. revealed in their study that patients with cerebral venous thrombosis had higher levels of homocysteine with low vitamin B_12_ levels as compared to controls [15]. Cantu *et al*. found that there is an independent association of low levels of folic acid, vitamin B_12_ as well as Hhcy with risk of cerebral venous thrombosis and mechanisms other than Hhcy should be analyzed for cerebral venous thrombosis risk [16]. There are anecdotal case reports suggesting correlation between Hhcy, vitamin B_12_ deficiency and cerebral venous thrombosis, deep vein thrombosis and acute porto-mesenteric vein thrombosis [17–19]. Ming-Ching Shen *et al.* reported that Hhcy caused deep vein thrombosis in a vitamin B_12_-deficient metformin-treated patient with diabetes [20]. There are conflicting reports in the literature about the association of Hhcy, B_12_ deficiency and cerebral venous thrombosis; however, most of the studies pointed to a significant association.

## Conclusion

We conclude that Hhcy is an independent risk factor for cerebral venous thrombosis in patients with cobalamin deficiency, especially those who follow a strict vegetarian diet. Hhcy can be easily reversed with vitamin supplementation, cobalamin and folic acid. Although there are conflicting reports in the literature about the association between Hhcy, B_12_ deficiency and cerebral venous thrombosis, in our view homocysteine levels should be checked in all patients with cerebral venous thrombosis for whom there is no other obvious predisposing factor for venous thrombosis. Further studies with large sample size are required to analyze the effect of Hhcy and low vitamin B_12_ on the risk of cerebral venous thrombosis.
